# Supplementary figures and images for: Co-occurrence of ST412 Klebsiella pneumoniae isolates with hypermucoviscous and non-mucoviscous phenotypes in a short-term hospitalized patient
Source: mSystems. 2024 Jun 21;9(7):e00262-24. doi: 10.1128/msystems.00262-24 (PMC11265266; doi:10.1128/msystems.00262-24)

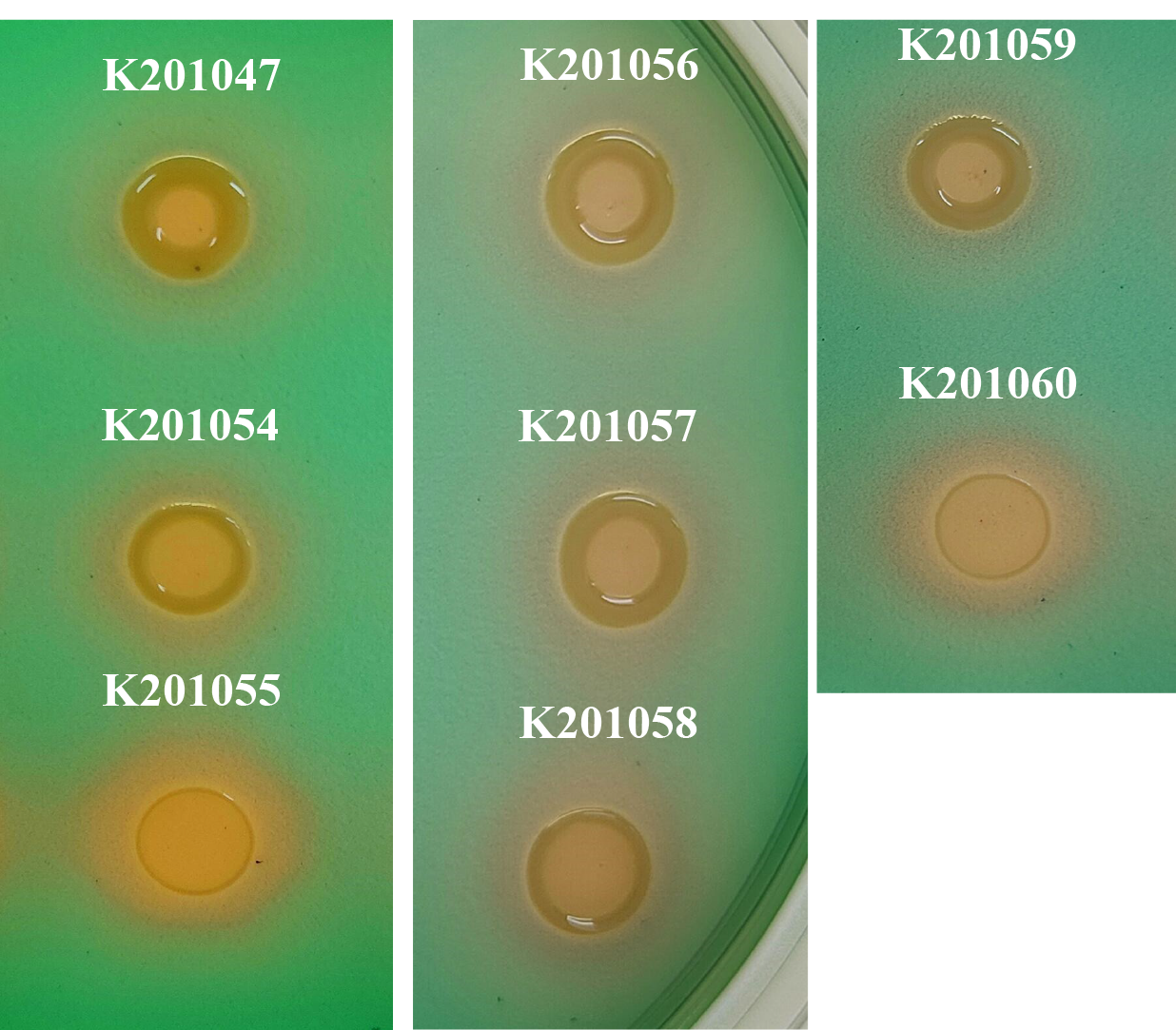

Supplement: Fig. S1 — Production of siderophores. [file msystems.00262-24-s0001.tif]

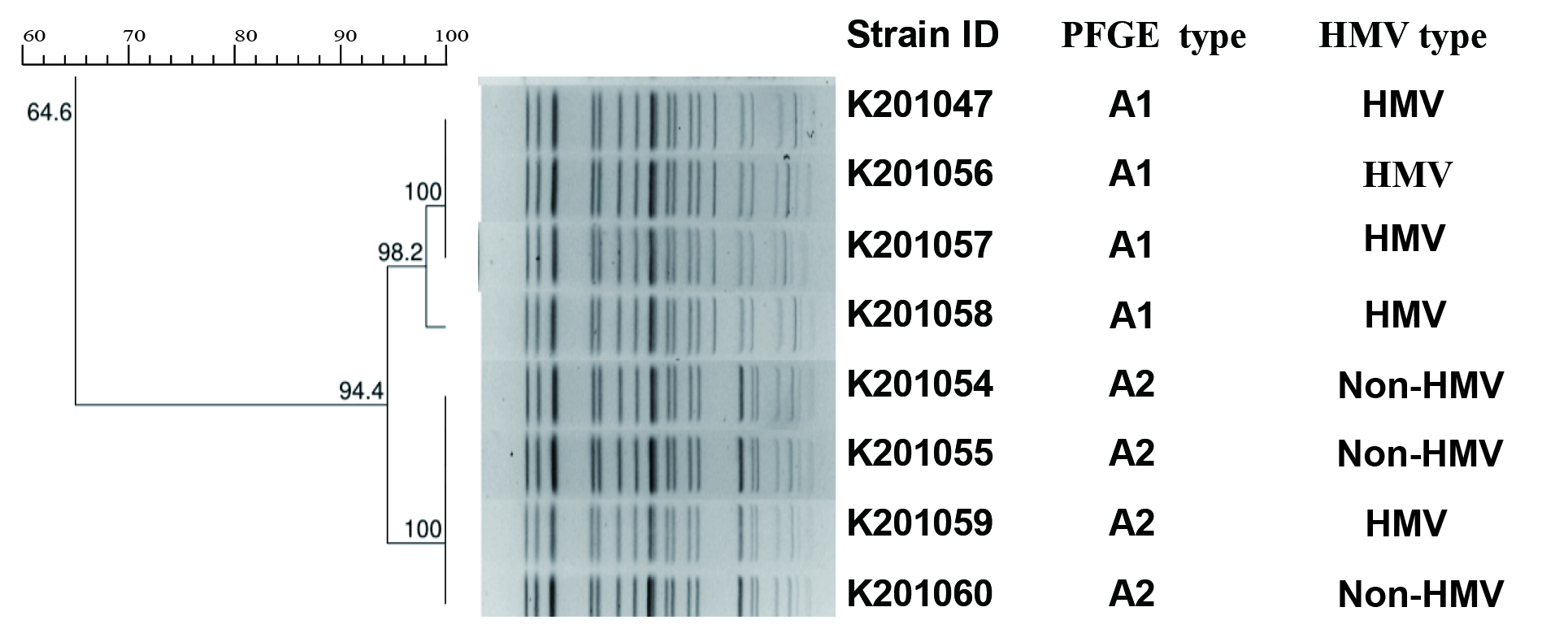

Supplement: Fig. S2 — PFGE cluster analysis. [file msystems.00262-24-s0002.tif]

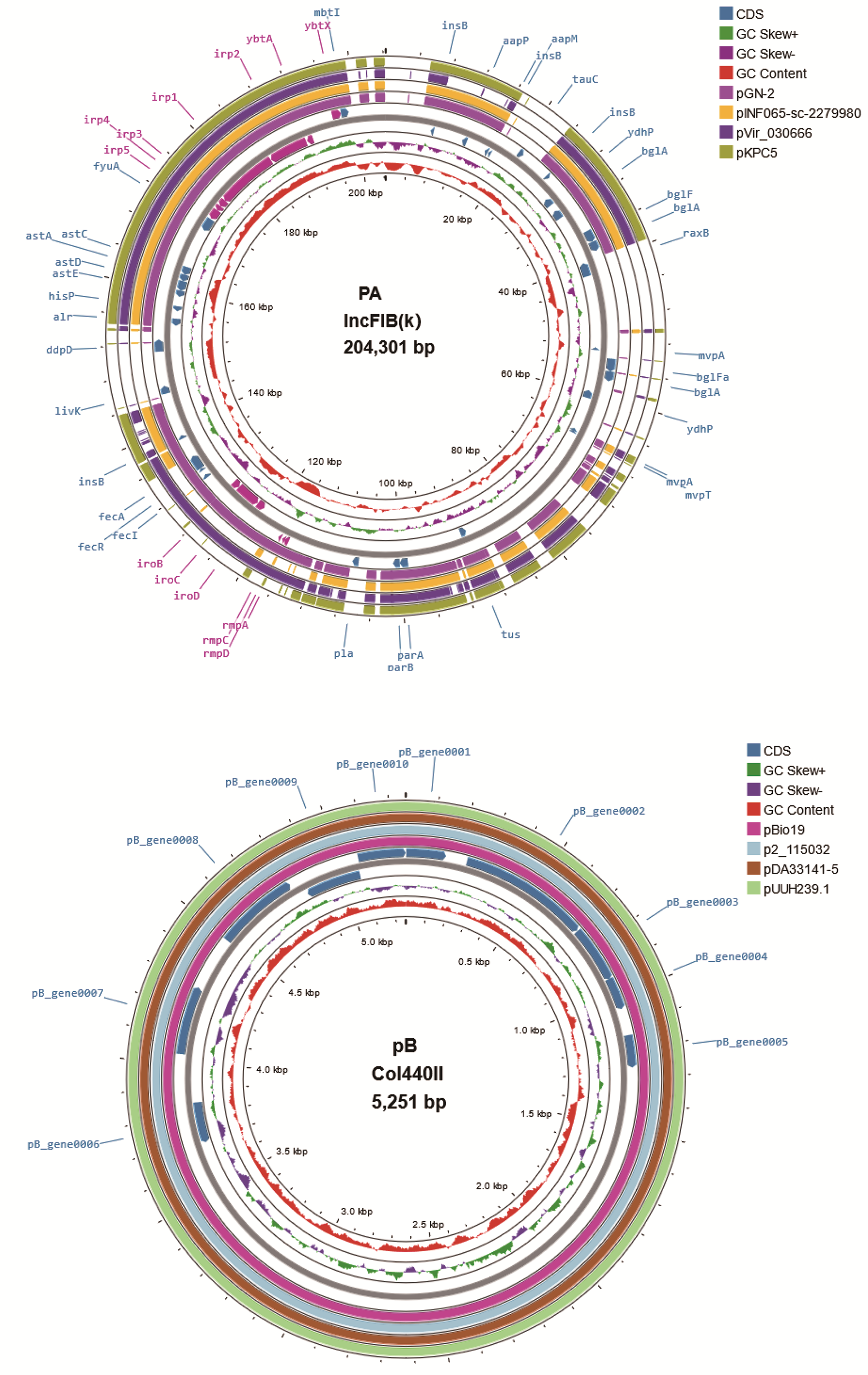

Supplement: Fig. S3 — Circular map of the plasmid. [file msystems.00262-24-s0003.tif]
