# Supplementary material for: Co-occurrence of ST412 Klebsiella pneumoniae isolates with hypermucoviscous and non-mucoviscous phenotypes in a short-term hospitalized patient
Source: mSystems. 2024 Jun 21;9(7):e00262-24. doi: 10.1128/msystems.00262-24 (PMC11265266; doi:10.1128/msystems.00262-24)
Supplement: Legends — Supplemental legends. [file msystems.00262-24-s0004.docx]

**Supplementary Material**

**Supplementary Figures**

**Fig.S1.** CAS plates were used to measure the production of siderophores. Orange halos indicate siderophore secretion.

**Fig.S2.** PFGE cluster analysis showed that the 8 isolates could be classified into 2 subtypes (A1 and A2).

**Fig.S3**. Circular map of the plasmid pA and plasmid pB in eight isolates and comparative genomics analysis with similar plasmids. The innermost rings show plasmids genome position (kbp = kilo base pairs); CDS region(blue); GC skew+(green); CG skew-(purple); GC content(red). The remaining rings of plasmid A (from 5 to ring 8) correspond to the genomes of pGN-2, pINF065-sc-2279980, pKPC5 and pVir_030666. The remaining rings of plasmid B (from 5 to ring 8) correspond to the genomes of pBio19, p2_115032, pDA33141-5 and pUUH239.1. Gene annotation. Red, virulence-related genes.

**Supplementary Tables**

**Table S1.** Antibiotic susceptibilities of eight isolates (mg/L) ^a^

**Table S2.** Significant transcriptional changes in GO and KEGG.

**Table S3.** Primers used in this study.

**Supplementary Data**

**Data S1.** Raw data for natural sedimentation assay

**Data S2.** Raw data for natural sedimentation experiment of recombinant strain

**Data S3.** Raw data for quantification of capsule

**Data S4.** Raw data for biofilm assays

**Data S5.** Raw data for siderophores secretion assays

**Data S6.** Raw data for serum resistance assay

**Data S7.** Raw data for mice infection experiments
