# Supplementary material for: Co-occurrence of ST412 Klebsiella pneumoniae isolates with hypermucoviscous and non-mucoviscous phenotypes in a short-term hospitalized patient
Source: mSystems. 2024 Jun 21;9(7):e00262-24. doi: 10.1128/msystems.00262-24 (PMC11265266; doi:10.1128/msystems.00262-24)
Supplement: Supplemental Material — Raw data for the individual isolates. [file msystems.00262-24-s0005.doc]

**Data S1**

Raw data for natural sedimentation assay

|  | OD600-supernatant/OD600-total | | |
| --- | --- | --- | --- |
| K201047 | 0.42 | 0.29 | 0.31 |
| K201054 | 0.42 | 0.44 | 0.41 |
| K201055 | 0.16 | 0.11 | 0.10 |
| K201056 | 0.46 | 0.59 | 0.50 |
| K201057 | 0.57 | 0.58 | 0.57 |
| K201058 | 0.54 | 0.53 | 0.52 |
| K201059 | 0.61 | 0.62 | 0.68 |
| K201060 | 0.11 | 0.12 | 0.08 |

**Data S2**

Raw data for natural sedimentation experiment of recombinant strain

|  | OD600-supernatant/OD600-total | | |
| --- | --- | --- | --- |
| K201054 | 0.46 | 0.51 | 0.47 |
| K201054-p*rmpA* | 1.16 | 1.26 | 1.17 |
| K201055 | 0.00 | 0.01 | 0.01 |
| K201055-p*wbaP* | 0.87 | 0.89 | 0.87 |
| K201060 | 0.01 | 0.01 | 0.01 |
| K201060-p*wbaP* | 0.75 | 0.59 | 0.55 |

**Data S3**

Raw data for quantification of capsule

|  | CPS (ug/1*10^9^cfu) | | |
| --- | --- | --- | --- |
| K201047 | 93.01 | 94.01 | 95.51 |
| K201054 | 81.43 | 78.01 | 81.01 |
| K201055 | 29.81 | 28.57 | 29.39 |
| K201056 | 104.42 | 110.22 | 107.80 |
| K201057 | 96.01 | 98.03 | 100.55 |
| K201058 | 73.47 | 73.92 | 77.11 |
| K201059 | 97.57 | 102.65 | 103.08 |
| K201060 | 43.01 | 39.74 | 42.54 |

**Data S4**

Raw data for biofilm assays

|  | Biofilm (OD_600_) | | |
| --- | --- | --- | --- |
| K201047 | 0.221 | 0.226 | 0.171 |
| K201054 | 0.430 | 0.485 | 0.426 |
| K201055 | 0.831 | 0.834 | 0.773 |
| K201056 | 0.229 | 0.210 | 0.160 |
| K201057 | 0.278 | 0.266 | 0.160 |
| K201058 | 0.308 | 0.303 | 0.315 |
| K201059 | 0.249 | 0.258 | 0.291 |
| K201060 | 0.850 | 0.942 | 0.854 |

**Data S5**

**Raw data for siderophores secretion assays**

|  | Orange halo (mm^2^) | | |  |
| --- | --- | --- | --- | --- |
| K201047 | 63.6 | 70.88 | 56.74 |  |
| K201054 | 78.5 | 70.88 | 63.6 |  |
| K201055 | 103.8 | 95 | 95 |  |
| K201056 | 78.5 | 78.5 | 80.11 |  |
| K201057 | 63.60 | 70.88 | 56.74 |  |
| K201058 | 78.50 | 70.88 | 95.00 |  |
| K201059 | 63.62 | 63.62 | 70.88 |  |
| K201060 | 113.00 | 122.70 | 95.00 |  |

**Data S6**

Raw data for serum resistance assay

|  | 0h (survival rate %) | | | 1h (survival rate %) | | | 2h (survival rate%) | | | 3h (survival rate %) | | |
| --- | --- | --- | --- | --- | --- | --- | --- | --- | --- | --- | --- | --- |
| K201047 | 100.0 | 100.0 | 100.0 | 5.9 | 20.0 | 4.5 | 0.0 | 6.0 | 0.0 | 0.0 | 0.0 | 0.0 |
| K201054 | 100.0 | 100.0 | 100.0 | 9.1 | 11.4 | 0.0 | 18.2 | 0.0 | 0.0 | 0.0 | 0.0 | 0.0 |
| K201055 | 100.0 | 100.0 | 100.0 | 146.1 | 168.6 | 228.6 | 3.2 | 27.5 | 7.9 | 0.0 | 0.0 | 1.6 |
| K201056 | 100.0 | 100.0 | 100.0 | 12.8 | 24.4 | 25.0 | 29.8 | 3.0 | 0.0 | 0.0 | 0.0 | 0.0 |
| K201057 | 100.0 | 100.0 | 100.0 | 6.4 | 3.0 | 18.8 | 0.0 | 0.0 | 0.0 | 0.0 | 0.0 | 0.0 |
| K201058 | 100.0 | 100.0 | 100.0 | 1.9 | 0.0 | 0.0 | 0.0 | 0.0 | 0.0 | 0.0 | 0.0 | 0.0 |
| K201059 | 100.0 | 100.0 | 100.0 | 12.3 | 3.7 | 48.9 | 5.3 | 0.0 | 0.0 | 5.3 | 0.0 | 0.0 |
| K201060 | 100.0 | 100.0 | 100.0 | 261.0 | 220.7 | 168.0 | 18.75 | 0.0 | 0.0 | 6.25 | 0.0 | 0.0 |

**Data S7**

Raw data for mice infection experiments

|  | Liver (Log_10_CFU/g) | | | | Spleen (Log_10_CFU/g) | | | | Kidney (Log_10_CFU/g) | | | |
| --- | --- | --- | --- | --- | --- | --- | --- | --- | --- | --- | --- | --- |
| K201047 | 7.08 | 7.19 | 7.09 | 7.02 | 7.72 | 7.92 | 7.76 | 7.75 | 7.20 | 7.29 | 7.31 | 7.36 |
| K201054 | 3.78 | 4.00 | 4.62 | 4.87 | 4.14 | 4.64 | 5.16 | 5.06 | 3.90 | 3.90 | 4.62 | 5.13 |
| K201055 | 4.02 | 2.80 | 2.72 | 2.70 | 4.29 | 4.21 | 4.42 | 3.94 | 3.74 | 2.82 | 2.98 | 3.09 |
| K201056 | 6.97 | 6.80 | 4.69 | 6.98 | 7.58 | 7.61 | 5.62 | 7.59 | 7.47 | 6.67 | 4.40 | 7.30 |
| K201057 | 7.02 | 7.16 | 7.07 | 7.03 | 7.61 | 7.45 | 8.32 | 7.52 | 7.20 | 7.32 | 7.13 | 7.23 |
| K201058 | 6.75 | 6.26 | 5.53 | 4.56 | 7.76 | 7.69 | 5.21 | 6.12 | 7.26 | 7.32 | 5.70 | 5.29 |
| K201059 | 7.03 | 6.88 | 6.94 | 6.87 | 7.36 | 7.78 | 7.43 | 7.39 | 7.08 | 7.29 | 7.33 | 7.30 |
| K201060 | 3.56 | 3.32 | 3.03 | 3.07 | 4.85 | 4.37 | 4.61 | 4.05 | 3.29 | 3.52 | 3.19 | 3.18 |
