# Supplementary material for: Co-occurrence of ST412 Klebsiella pneumoniae isolates with hypermucoviscous and non-mucoviscous phenotypes in a short-term hospitalized patient
Source: mSystems. 2024 Jun 21;9(7):e00262-24. doi: 10.1128/msystems.00262-24 (PMC11265266; doi:10.1128/msystems.00262-24)
Supplement: Table S1 — Antibiotic susceptibilities of eight isolates. [file msystems.00262-24-s0006.docx]

**Table**

**Table S1 Antibiotic susceptibilities of eight isolates (mg/L) ^a^**

| Isolate | MIC (mg/L) | | | | | | |
| --- | --- | --- | --- | --- | --- | --- | --- |
|  | CTX | CAZ | MEM | CIP | TET | PB | C |
| K201047 | 0.5 | 2 | 0.5 | 0.25 | 2 | 0.5 | 4 |
| K201054 | 0.5 | 2 | 0.5 | 0.25 | 2 | 0.5 | 4 |
| K201055 | 0.5 | 2 | 0.5 | 0.25 | 2 | 0.5 | 4 |
| K201056 | 0.5 | 2 | 0.5 | 0.25 | 2 | 0.5 | 4 |
| K201057 | 0.5 | 2 | 0.5 | 0.25 | 2 | 0.5 | 4 |
| K201058 | 0.5 | 2 | 0.5 | 0.25 | 2 | 0.5 | 4 |
| K201059 | 0.5 | 2 | 0.5 | 0.25 | 2 | 0.5 | 4 |
| K201060 | 0.5 | 2 | 0.5 | 0.25 | 2 | 0.5 | 4 |

^a^ CTX, cefotaxime; CAZ, ceftazidime; CIP, ciprofloxacin; TET, tetracycline; PB, polymyxin B; C, chloramphenicol.
