# Supplementary material for: Co-occurrence of ST412 Klebsiella pneumoniae isolates with hypermucoviscous and non-mucoviscous phenotypes in a short-term hospitalized patient
Source: mSystems. 2024 Jun 21;9(7):e00262-24. doi: 10.1128/msystems.00262-24 (PMC11265266; doi:10.1128/msystems.00262-24)
Supplement: Table S3 — Primers. [file msystems.00262-24-s0008.docx]

**Table**

Table S3. Primers used in this study

| Primer | Sequences (5’—3’) |
| --- | --- |
| Primers for complements assays | |
| *rmpA*-KpnI-F | CGGGGTACCTCTATTCATCGCTTTTC |
| *rmpA*-XbaI-R | CTAGTCTAGATGTAACCAACGACTTTC |
| *wbaP*-KpnI-F | CGGGGTACC GTTGGAAGATAAGGGAGTT |
| *wbaP*-ScaI-R | CGAGCTCGTGAGAAGAAAAAGAGAGCG |
| *wbaP*-F | CCTCTCGGGATGGACT |
| *wbaP-*R | CCCTTTGATAACTACGCCTGT |
| *rmpA-*F | AATGGATGTGGCTTGAC |
| *rmpA-*R | ATTGCAGCACTGCTTGT |
| Primers for qpcr assays | |
| q-*pduQ*-F | TACCGCCCACCATCACAGC |
| q*-pduQ-R* | CCCTGACGAATCGCAACG |
| q***-****pflD-F* | CGTTTACCTACGCCAAAGATG |
| q*-pflD-R* | TGACGTTGAGATGCTGTCCC |
| q*-aceB-F* | ATCAGTTGCAGCCCAATCC |
| q*-aceB-R* | CGCCTGGTAGTTATGAAAGAAA |
| q*-puuA-F* | CGAAACCTACCCTGATACCC |
| q*-puuA-R* | TCCACCACATTGCCGAGA |
| q*-puuD-F* | GTCCCATCAGGTTGAGGTTC |
| q*-puuD-R* | CGTATTCGCTACTGTTCCATTC |
| q*-fadD-F* | CCTGACCGCTATCAATCCC |
| q-*fadD*-R | ATGCCAAACAGAGCCACC |
| q-*fimI*-F | CGCAATACCGATCCCACT |
| q-fimI-R | ACAGCAACCCAATCCCTT |
| q-fimC-F | TCCGCTACGGCTAAAGGT |
| q-fimC-R | CAGGATCGGGAAAGTCTGTT |
| q-fimG-F | CTTTAGCCTGATCGGTGCG |
| q-fimG-R | TGCCGTCCTCGTTCTGC |
| q-cysP -F | GGAGCGACCACCACCTT |
| q-cysP -R | GGAACTCCGCCAGAATG |
| q-yaiY -F | AGCCATGTCGCCTACGG |
| q-yaiY -R | AAGGAAAGGGATCAGACCAAA |
| q-ypeC-F | AGCCAGCACGATAGCCG |
| q-ypeC-R | GGCGTCGTCACTATGAATGG |
| q-cydA-F | TGGTGCTTCAGGGTATTCAG |
| q-cydA-R | GGCGATCCGTAAGATGTTCT |
| q-yciW-F | GGCGCAAATCACGCAATA |
| q-yciW-R | GCCTGGAAGCCGACGAAT |
| q-tktA-F | CTCGGTGGTCTCGGTCATC |
| q-tktA-R | CTTTCAGTTCCTCCAGTGCC |
| q-metF-R | AAGGGCTGGATAACGACG |
| q-metF-R | GGTTCAGGGTGTAGAAGTGGA |

Underline indicates the restriction sites
